# Highly Fermentable Fiber Alters Fecal Microbiota and Mitigates Swine Dysentery Induced by *Brachyspira hyodysenteriae*

**DOI:** 10.3390/ani11020396

**Published:** 2021-02-04

**Authors:** Emma T. Helm, Nicholas K. Gabler, Eric. R. Burrough

**Affiliations:** 1 Department of Animal Science, Iowa State University, Ames, IA 50011, USA; ethelm@iastate.edu (E.T.H.); ngabler@iastate.edu (N.K.G.); 2 Department of Veterinary Diagnostic and Production Animal Medicine, Iowa State University, Ames, IA 50011, USA

**Keywords:** pig, nutrition, fermentable fiber, insoluble fiber, *Brachyspira hyodysenteriae*, microbiota

## Abstract

**Simple Summary:**

Dietary manipulation to prevent disease is essential to reduce antimicrobial usage in the swine industry. This study aimed to evaluate whether replacing lowly fermentable fibers with highly fermentable fibers would mitigate disease during a 42 day *Brachyspira hyodysenteriae* challenge. Pigs fed the highly fermentable diet had improved growth performance compared with those fed diets of lower fermentability and had near absence of clinical swine dysentery. Further, several microbial genera were altered by dietary manipulation, bacteria that may be synergistic or antagonistic to *Brachyspira hyodysenteriae*. Taken together, this study demonstrates that replacing lowly fermentable fiber with highly fermentable fibers mitigates disease during *Brachyspira hyodysenteriae* challenge and may help reduce antimicrobial usage in treatment and control of this pathogen.

**Abstract:**

*Brachyspira hyodysenteriae* is an etiological agent of swine dysentery (SD). Diet fermentability plays a role in development of SD, but the mechanism(s) of action are largely unknown. Thus, this study aimed to determine whether replacing lowly fermentable fiber with highly fermentable fiber would mitigate a 42 d *B. hyodysenteriae* challenge. Thirty-nine barrows were allocated to dietary treatment groups: (1) 20% corn distillers dried grain with solubles (DDGS), 0% beet pulp (BP) or resistant starch (RS; lowly fermentable fiber (LFF)); (2) 10% DDGS, 5% BP, 5% RS (medium fermentable fiber (MFF)); and (3) 0% DDGS, 10% BP, 10% RS (highly fermentable fiber (HFF)). On day post inoculation 0, pigs were inoculated with *B. hyodysenteriae*. Overall, 85% LFF pigs developed clinical SD, 46% of MFF pigs developed SD, and 15% of HFF pigs developed SD (*p* < 0.05). Overall average daily gain (ADG) differed among all treatments (*p* < 0.001), with LFF pigs having the lowest ADG. For HFF pigs, ADG was 37% greater than LFF pigs (*p* < 0.001) and 19% greater than MFF pigs (*p* = 0.037). The LFF diet had greater relative abundance of *Shuttleworthia* and *Ruminococcus torques*. Further, microbiota of pigs that developed SD had enriched *Prevotellaceae*. Collectively, replacing DDGS with highly fermentable fiber reduced clinical SD, improved performance, and modulated fecal microbiota during *B. hyodysenteriae* challenge.

## 1. Introduction

Immune stress associated with pathogen infection remains one of the largest issues facing pork producers worldwide. One such pathogen, *Brachyspira hyodysenteriae*, is an etiological agent of swine dysentery (SD) in growing pigs [1]. This spirochete infects the cecum and colon of growing pigs and causes disease characterized by mucohemorragic diarrhea, dehydration, appetite reduction, and rapid weight loss [1]. Diet, specifically dietary fiber, is known to play a large role in the colonization of *B. hyodysenteriae* and the development of swine dysentery, but much is unknown regarding the mechanism of action behind this phenomenon. Further, there is disparity with regards to which specific fiber types prevent disease [2,3]. In general, it seems that lowly fermentable, insoluble fibers such as those found in corn distillers dried grains with solubles (DDGS) shorten the time to onset and increase the incidence of disease [4], limiting fermentation in the hindgut may decrease disease [2,5], and that highly fermentable, soluble fibers may increase or decrease disease incidence depending on the specific fiber type and base diet composition [2,3,6].

Several factors may be responsible for differences in disease expression due to dietary fiber. First, changes in mucin composition may alter the ability of *B. hyodysenteriae* to associate with and colonize the colonic mucosa. Dietary fiber increases mucus production and may alter mucin composition [7,8], which could increase susceptibility to *B. hyodysenteriae* challenge, as these components are chemoattractants for *B. hyodysenteriae*. In particular, *B. hyodysenteriae* is attracted to sialic acid residues on acidic mucins, which are highly abundant in pigs with clinical swine dysentery [9]. However, it is unclear whether dietary fiber increases sialic acid mucin production, and whether that allows for greater *B. hyodysenteriae* colonization.

Additionally, commensal microbial populations in the large intestine likely influence *B. hyodysenteriae* colonization and disease pathogenesis. Dietary fibers are largely resistant to small intestinal digestion and absorption but can be completely or partially fermented in the large intestine [10]. Insoluble fibers such as lignins and cellulose are poorly fermented by microbial populations, while more soluble fibers and resistant starches are highly fermented in the large intestine [11]. Specifically, these highly fermentable fibers promote lactic acid- and butyric acid-producing bacteria, such as *Bifidobacterium*, *Megasphaera*, and *Faecalibacterium* [12,13]. Conversely, diets high in insoluble fiber are associated with a reduction in lactic acid producers including *Lactobacillus* and *Megasphaera* [12,14]. Some of these bacterial genera may promote health in the face of *B. hyodysenteriae* challenge, as inoculated pigs that do not develop clinical disease have a higher abundance of *Lactobacillus* and *Bifidobacterium* than pigs that do develop clinical disease [15]. This has led researchers to hypothesize that highly fermentable fiber components may allow for greater abundance of microbial communities that prevent *B. hyodysenteriae* colonization and infection, while lowly fermentable fiber components may promote microbial communities that aid *B. hyodysenteriae* colonization and infection. However, a direct comparison between dietary fiber alterations and microbial communities before, during, and after challenge with *B. hyodysenteriae* is lacking.

We have previously demonstrated that replacing corn DDGS with highly fermentable sugar beet pulp and resistant potato starch delays onset and reduces severity of *B. hyodysenteriae* challenge [16]. However, longitudinal growth performance during the recovery phase of disease was not evaluated. Thus, the objective of this study was to determine whether replacing lowly fermentable corn DDGS with more highly fermentable fibers (sugar beet pulp and resistant starch), would mitigate clinical disease and improve pig growth performance during a 6 week *B. hyodysenteriae* challenge. Further, we evaluated fecal microbiota at multiple time points over the course of the challenge study to determine whether diet-induced shifts in microbiota could explain differences in disease susceptibility.

## 2. Materials and Methods

The Iowa State University Institutional Animal Care and Use Committee (IACUC protocol #19-170) approved all animal procedures, which adhered to the ethical and humane use of animals for research.

### 2.1. Animals, Housing, and Experimental Design

A total of 39 barrows (29.4 ± 4.0 kg body weight (BW); Camborough (1050) × 337, Hendersonville, TN) were selected randomly and confirmed negative for *B. hyodysenteriae* and *B. hampsonii* via selective anaerobic fecal culture of individual fecal swabs at the Iowa State University Veterinary Diagnostic Laboratory (ISU VDL) using routine methodologies. Pigs were then assigned to individual pens and allotted to treatment groups as follows (n = 13 pigs/treatment(trt): (1) 20% DDGS, 0% BP or RS (low fermentable fiber, (LFF)), (2) 10% DDGS, 5% BP, 5% RS (medium fermentable fiber (MFF)), and (3) 0% DDGS, 10% BP, 10% RS (high fermentable fiber (HFF)). Dietary treatments were initiated 14 d prior to the *B. hyodysenteriae* challenge to allow for dietary adaptation. Both diets met or exceeded all National Research Council [17] requirements for this size pig, were ad libitum fed throughout the experiment, and contained no antibiotics (Table 1).

A feed sample from all complete diets was collected for analysis. Feed samples were ground through a 1-mm screen (Model ZM1; Retsch Inc., Newton, PA, USA) and dietary proximate analysis was performed. Diet samples were analyzed for dry matter (AOAC method 930.15), gross energy via bomb calorimetry (Oxygen Bomb Calorimetry 6200; Parr Instruments, Moline, IL, USA), and nitrogen using the TruMac N (Leco Corporation, St. Joseph, MO, USA). Diet samples were sent to the University of Missouri Experimental Station Chemical Laboratories (Colombia, MO, USA) for analysis of acid detergent fiber, neutral detergent fiber, insoluble fiber, and total dietary fiber using official Association of Official Analytical Chemists methods [18]. Dietary resistant starch was evaluated using the Megazyme Resistant Starch Assay Kit (Megazyme Ltd., Bray, Ireland).

On day post inoculation (dpi) 0, all pigs were inoculated with *B. hyodysenteriae* B204. Briefly, following a 12–18 h fast, all pigs received 1 dose of agar slurry inoculum (60 mL/dose; approx. 1.6 × 10^6^ colony forming units/mL) administered via gastric gavage. Individual feed disappearance and BW were recorded for each pig on dpi −14, 0, 7, 14, 21, 28, 35, and 42. From these recordings, individual pig average daily gain (ADG), average daily feed intake (ADFI), and feed efficiency (gain:feed; G:F) were calculated.

Fecal swabs were collected on all pigs at dpi 4, 8, 12, 14, 15, 20, 28, 35, and 42. Swabs were submitted to the ISU VDL to be selectively cultured for *B. hyodysenteriae* using routine methodology of the laboratory. These dates were chosen to closely monitor fecal shedding during peak infection (dpi 4–20), and then occasionally surveil for the rest of the experimental period. Fecal consistency was scored each morning by the same individual blinded to dietary treatments. Fecal consistency was scored based on the following scale: 0 if normal, 1 if soft but formed, 2 if semisolid, and 3 if liquid to watery. An additional 0.5 point was added for the presence of blood and/or mucus, with a maximum score of 4. Clinical swine dysentery was defined by a score of 4 (watery diarrhea containing both blood and mucus).

To assess core body temperatures, all pigs were microchipped with a bio-sensor chip (LifeChip^®^ with Bio-Thermo Technology, Destron Fearing, Eagan, MN, USA) in the neck muscle on dpi −7. All pigs were scanned daily with a Global Pocket Reader™ Plus universal reader (Destron Fearing, Eagan, MN, USA) between 700 and 900 h.

On dpi 42, all pigs were euthanized for tissue collection. The apex of the spiral colon was fixed in formalin and then trimmed, processed, embedded, and sectioned at the ISU VDL. Cecum and spiral colon contents were collected into 50 mL conical tubes and a portable pH probe (ThermoFisher Scientific, Waltham, MA, USA) was used to evaluate pH.

### 2.2. Histopathology

Sialomucins and sulfomucins were identified by treating deparaffinized, formalin-fixed spiral colon sections with Alcian blue (pH 2.5) and high iron diamine at the ISU VDL according to Curry, et al. [19]. Briefly, sections were incubated with high iron diamine, rinsed, incubated with Alcian blue, rinsed, dehydrated, and then mounted. Whole colon cross-sections were scanned at 4× using a DP80 Olympus Camera (Olympus Scientific, Waltham, MA, USA) mounted on an OLYMPUS BX 53/43 microscope (Olympus Scientific, Waltham, MA, USA) with a motorized stage. Regions that contained approximately 5 well-orientated crypts were imaged at 20×. Approximately 3–4 images were taken per cross-section to acquire at least 12 well-orientated crypts. Image analysis was performed using HALO image analysis software (HALO™, Indica Labs, Inc., Corrales, NM, USA). Individual crypts were outlined as the region of interest, high iron diamine- and Alcian blue-positive goblet cells were enumerated by HALO, and data were presented as the number of high iron diamine- or Alcian blue-positive goblet cells per 10,000 μm^2^.

### 2.3. Fecal DNA Extraction and 16S Library Preparation

Individual fresh fecal samples were collected on all pigs at dpi 0, 12, and 42. Fecal samples were snap-frozen in liquid nitrogen and stored at −80 °C until DNA extraction. Fecal samples from 10 pigs per treatment were selected and submitted to the ISU VDL and processed for DNA extraction. Samples were randomly chosen from the MFF and HFF diets, for the LFF diet antibiotic treated pigs were excluded from analysis. Deoxyribonucleic acid was extracted using the Qiagen DNeasy PowerSoil Kit and PowerMag DNA Isolation Kit (Qiagen, Germantown, MD, USA) according to the manufacturer’s recommendations. DNA concentrations and purity were determined using a spectrophotometer. DNA was stored at −80 °C prior to further processing.

After extraction, DNA was sequenced at the ISU VDL using the Ilumina MiSeq platform as previously described [20]. Briefly, the V4 regions of the 16S rRNA gene were sequenced with the Illumina MiSeq sequencing platform using v2 MiSeq cartridges (Illumina, Inc., San Diego, CA, USA). This produced 2 × 250 bp paired end reads. DNA was amplified by using the 515f/806r primer set (Forward V4: GTGCCAGCMGCCGCGGTAA; Reverse V4: GGACTACHVGGGTWTCTAAT). Primers were designed based on the V4 primers, illumina adapter sequences, and multiplex indices. Polymerase chain reaction amplification of extracted DNA was performed with Schloss lab indices and AccuPrime™ Pfx SuperMix (Thermofisher Scientific, Waltham, MA, USA; Cat: 12344-040). Products of PCR were run on Qiagen Qiaxcel to confirm amplification. Libraries were cleaned with Agencourt AMPure XP beads (Beckman Coulter^®^, Pasadena, CA, USA), and library quantification was performed using the Qubit™ dsDNA HS Assay Kit (Invitrogen, Carlsbad, CA, USA) with a Qubit^®^ 2.0 Fluorometer (Thermofisher Scientific, Waltham, MA, USA). Libraries were pooled to single tube to ensure each library had the same final concentrations.

The pooled library was sequenced with 15% PhiX, at final concentration of 4pM, using MiSeq Reagent Nano Kit v2 (Illumina, Inc., San Diego, CA, USA) for a 500 cycle (2 × 250 bp) run. Custom sequencing primers for read 1 (5′-TATGGTAATTGTGTGCCAGCMGCCGCGGTAA-3′); Read 2 (5′-AGTCAGTCAGCCGGACTACHVGGGTWTCTAAT-3′), and index read (5′-ATTAGAWACCCBDGTAGTCCGGCTGACTGACT-3″) were used. Demultiplexing of raw data was performed based on dual indices and two fastq files were generated for each sample.

Upstream analysis was performed in QIIME 2 [21]. Primer sequence were removed from reads using the cutadapt QIIME 2 plugin. The DADA2 pipeline [22] was used to denoise the reads and construct feature table and representative sequences for amplicon sequence variants (ASVs or features). Operational taxonomic units (OTUs) were then assigned to ASVs using a Naive-Bayes approach implemented in the classify-sklearn against the SILVA database v132 [23]. Rare (frequency < 50) ASVs; ASVs classified to eukaryota, mitochondria and chloroplast; and ASVs that failed to be classified to at least phylum level were removed. Feature table and representative sequences were adjusted accordingly. Filtered ASVs were used for downstream analysis. Raw sequence data were deposited in the Sequence Read Archive (accession #PRJNA678760).

### 2.4. Bioinformatics

The linear discriminant analysis effect size (LEfSE) method (Galaxy v1.0) was used to determine biological effect size of differentially abundant OTUs [24]. Only OTUs present in at least 25% of samples were used for analysis. Samples from dpi 0, 12, and 42 were analyzed separately in LEfSE for dietary treatment effects. Samples from dpi 0 were further analyzed to evaluate differences in pigs that did or did not develop clinical disease, using dietary treatment as subclass to avoid confounding effects of diet.

### 2.5. Statistical Analysis

Statistical analysis of clinical parameters, weekly growth performance, digesta pH, and histochemistry data was performed in SAS 9.4 (SAS Institute Inc., Cary, NC, USA). The following mixed model was fitted to all parameters:

Y_ijk_ = μ + Diet_i_ + e_ij_ (1)

wherein Y_ij_ = the phenotype measured on animal j; µ = the overall mean; Diet_i_ = effect of diet (fixed effect; LFF, MFF, HFF); and e_ij_ = error term of animal j subjected to treatment i, e_ij_ ~ N(0, σ_e_^2^). The least squares (LS) means statement was used to produce LS means were produced using the LS means statement and the pdiff option was used to generate differences in LSmeans. Daily core body temperatures and 0–42 dpi body weights were analyzed using the same model as above with the inclusion of a repeated measures statement. Histochemistry count data were evaluated using the GLIMMIX procedure and a Poisson distribution. All data are presented as LS means with a pooled standard error of the mean (SEM). Fisher’s exact tests in the FREQ procedure were used to determine whether treatment contributed to the number of pigs that developed clinical SD or that were positive for *B. hyodysenteriae* via fecal culture. Log rank tests were performed using LIFETEST procedure to determine whether treatment contributed to the number of days until pigs developed clinical SD.

Diversity metrics and PCoA ordination plots were generated using the Phyloseq package (v1.30.0) [25] in RStudio v1.2.5033 and QIIME 2. Statistical pair-wise comparisons were performed to test the alpha and beta diversity group significance among treatments. Differential abundance analysis was performed using the GLIMMIX procedure in SAS. Taxa existing in fewer than 25% of the samples were discarded. Counts were analyzed assuming a negative binomial distribution, logarithmic link function, and offset to the natural log of library size. Means separation was performed using the DIFF option to perform pairwise comparisons. Adjustment of *p*-values was performed using the Benjamini and Hochberg method [26] with a false discovery rate controlled at 5%. Differences were considered significant when *p* < 0.05 and a tendency when *p* ≤ 0.05, *p* ≤ 0.10.

## 3. Results

### 3.1. Clinical Observations, Diagnostics, and Growth Performance

Loose stools containing blood and mucus were first observed at dpi 7 for LFF pigs, dpi 9 for MFF pigs, and dpi 11 for HFF pigs (Table 2). Antimicrobial treatment was required for 4 pigs due to disease severity; all fed the LFF diet. Pigs requiring treatment were treated with lincomycin hydrochloride via intramuscular injection for 5 d, either dpi 13–17 (3 pigs) or dpi 17–21 (1 pig). All antimicrobial treatments occurred after the first (dpi 0) and second (dpi 12) fecal collections for 16S rRNA sequencing, and three of these pigs were excluded from 16S rRNA microbial sequencing entirely. One pig on the MFF diet died from severe acute dysentery on dpi 10, this was accounted for in statistical analysis.

The median number of days it took pigs to develop clinical swine dysentery (loose stools containing both blood and mucus) differed among treatments (*p* < 0.001; Table 2), with LFF pigs taking 9 days and MFF pigs taking 20 days to develop clinical swine dysentery. As only 2 HFF pigs developed clinical swine dysentery, median days to develop disease was incalculable. Overall, the number of pigs with clinical swine dysentery at any point during the 42 day study differed among treatments (*p* = 0.002), with 85% of LFF pigs developing swine dysentery, 46% of MFF pigs having swine dysentery, and only 15% of HFF pigs ever having clinical swine dysentery. Further, the median days clinical disease was observed for each pig was reduced by the addition of highly fermentable fiber (*p* = 0.001). On average, the duration of clinical swine dysentery was 6 days for LFF pigs, 2 for MFF pigs, and 0 for HFF pigs due to the high number of pigs without clinical disease. These difference in clinical disease were not due to failure of *B. hyodysenteriae* to colonize as there were no differences in the number of pigs that tested positive for *B. hyodysenteriae* via fecal shedding at any point during the 42 day study (*p* = 0.588). A summary of fecal culture results can be found in Table S1. In general, 35 of the 38 pigs shed *B. hyodysenteriae* at some point during the study, indicating a high colonization rate.

Core body temperatures were assessed daily starting at dpi −7 (Figure S1). Overall, core body temperatures remained relatively stable over the duration of the study, barring a drop in temperature at dpi 0 after the overnight fast prior to inoculation. Post inoculation, an observable febrile response was not detected. Dietary treatment did influence core body temperatures throughout the experiment (*p* = 0.032), with pigs on more fermentable diets generally having higher body temperatures, although these differences were quite small.

In the 14-day pre-challenge period, neither ADG nor ADFI differed among treatments. However, G:F differed (*p* = 0.001; Table 3), with HFF pigs having greater G:F than both MFF (*p* = 0.040) and LFF (*p* = 0.001) pigs. At dpi 0, initial body weight did not differ among treatments (*p* = 0.623; Table 3).

**Figure 1:**
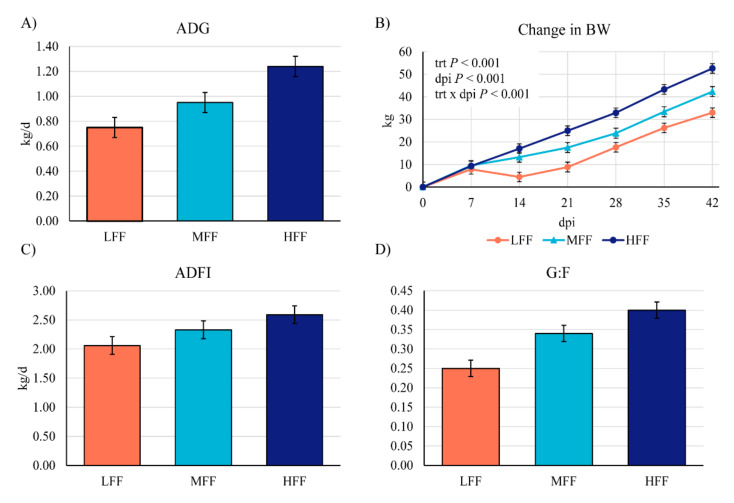
Overall (0–42 days post inoculation (dpi)) average daily gain, change in BW, average daily feed intake and feed efficiency (G:F) of pigs inoculated with *Brachyspira hyodysenteriae* and fed a lowly fermentable diet (LFF), an intermediate fermentability diet (MFF), and a highly fermentable diet (HFF).

From dpi 0–42, ADG differed among all treatments (*p* < 0.001), with LFF pigs having the lowest ADG (Figure 1). For MFF pigs, overall ADG was 22 % greater than LFF pigs (*p* = 0.009). For HFF pigs, overall ADG was 37% greater than LFF pigs (*p* < 0.001) and 19% greater than MFF pigs (*p* = 0.037; Figure 1). Overall ADFI was 20% greater in HFF pigs compared with LFF pigs (*p* = 0.013), MFF pigs being intermediate. Overall G:F was greater in HFF pigs compared with both MFF (13%, *p* = 0.001) and LFF (20%, *p* < 0.001) pigs. As a result of these performance differences, end body weight differed among treatments such that HFF pigs were the heaviest at dpi 42, weighing 11.3 kg more than MFF (*p* = 0.037) and 21.4 kg more than LFF (*p* < 0.001) pigs. Final (dpi 42) body weight tended to be greater in MFF pigs compared with LFF pigs (*p* = 0.062).

Differences in overall performance were primarily driven by changes in growth rates from dpi 0–14, when deviations to growth curves were observed in both MFF and LFF pigs (Figure 1). From dpi 0–7, ADG was reduced in LFF pigs compared with both MFF (*p* = 0.009) and HFF (*p* = 0.030) pigs, which did not differ from each other (Table 3). ADFI did not differ from dpi 0–7, thus G:F was reduced in LFF pigs compared with both MFF (*p* = 0.030) and HFF (*p* = 0.002) pigs. From dpi 8–14, ADG was greatly reduced in LFF pigs compared with both MFF (*p* = 0.031) and HFF (*p* < 0.001) pigs. Further, ADG tended to be reduced in MFF pigs compared with HFF pigs (*p* = 0.083). Similarly, from dpi 8–14 ADFI and G:F were reduced in LFF pigs compared with MFF and HFF pigs (*p* < 0.05 for all).

Fewer differences in performance were observed from dpi 15–42. From dpi 15–21, only ADFI differed among treatments, being reduced in LFF pigs compared with HFF pigs (*p* = 0.010). From dpi 22–28 and dpi 29–35, there were no differences in ADG, ADFI, or G:F. From dpi 36–42, ADG was greater in MFF pigs compared with LFF pigs (*p* = 0.042), HFF pigs intermediate. Similarly, ADFI was reduced in LFF pigs compared with both MFF (*p* = 0.016) and HFF (*p* = 0.031) pigs. Feed efficiency did not differ among treatments from dpi 36–42 (*p* < 0.10).

### 3.2. Digesta pH and Colon Goblet Cell Counts

Digesta pH in both the cecum and colon were evaluated at dpi 42 (Table 4). Cecum pH was greater in LFF pigs compared with both MFF (*p* = 0.047) and HFF pigs (*p* = 0.015), which did not differ from another. Numerically, colon pH values followed similar trends, but was not statistically different among treatments (*p* = 0.139).

**Figure 2:**
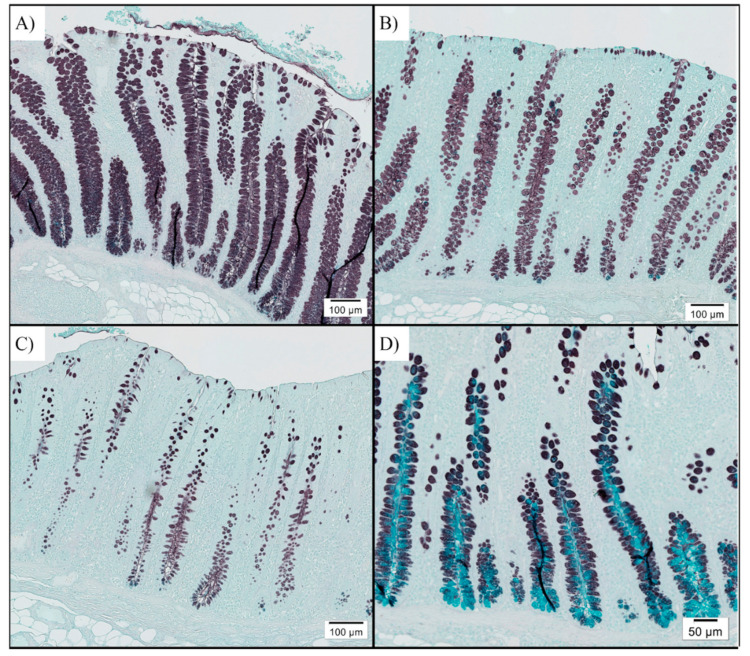
Representative high iron diamine (black stain) and Alcian blue (turquoise stain) histochemistry images taken at 20X to differentiate sulfomucins and sialomucins, respectively. Pigs were inoculated with *Brachyspira hyodysenteriae* and fed a (**A**) lowly fermentable diet (LFF), an (**B**) intermediate fermentability diet (MFF), and a (**C**) highly fermentable diet (HFF). Pigs were euthanized at days post inoculation (dpi) 42. In general, colon sections were dominated by sulfomucins, with very few goblet cells containing sialomucins being present unless pigs were still clinically afflicted at the time of necropsy (panel **D**). If sialomucin-positive cells were present, these were primarily limited to the base of the crypts.

Fixed colon tissues were mounted on slides and stained with high iron diamine and alcian blue to enumerate sulfomucin- and sialomucin-positive goblet cells, respectively. In general, colon samples were dominated by sulfomucin-positive goblet cells, suggesting disease resolution (Figure 2). Sialomucin-positive cells were sparse and limited to the base of the crypts if present at all. The number of sulfomucin-positive cells differed among treatments, with HFF pigs having fewer sulfomucin-positive goblet cells than LFF pigs (*p* = 0.032; Table 5). The number of sialomucin-positive goblet cells did not differ among treatments (*p* = 0.990).

### 3.3. Microbial Communities

**Figure 3:**
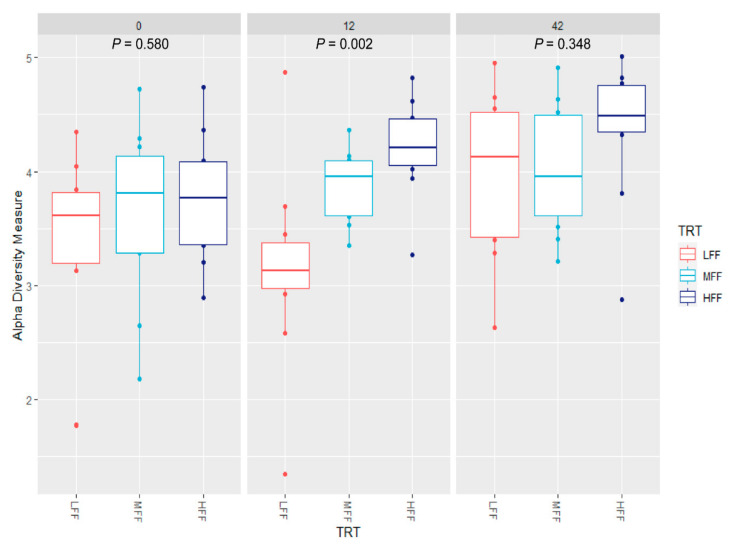
Shannon diversity boxplots of fecal microbial populations in pigs inoculated with *Brachyspira hyodysenteriae* and fed a lowly fermentable diet (LFF), an intermediate fermentability diet (MFF), and a highly fermentable diet (HFF) at days post inoculation 0, 12, or 42. TRT = treatment.

**Figure 4:**
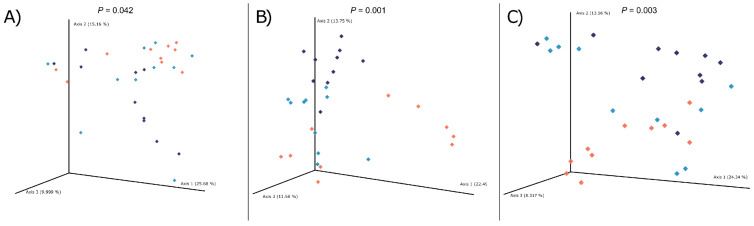
Beta diversity (Bray–Curtis dissimilarity plots) of fecal microbial populations in pigs inoculated with *Brachyspira hyodysenteriae* and fed a lowly fermentable diet (orange; LFF), an intermediate fermentability diet (turquoise; MFF), and a highly fermentable diet (navy blue; HFF) at days post inoculation (**A**) 0, (**B**) 12, or (**C**) 42.

At dpi 0, no differences in Shannon diversity were observed between treatment groups (treatment *p* = 0.580; Figure 3). Diet had a significant effect on beta diversity at dpi 0, with HFF pigs differing from LFF pigs (*p* = 0.009; Figure 4). At dpi 12, alpha diversity differed among all treatment groups (*p* ≤ 0.041 for all pairwise comparisons). For beta diversity at dpi 12, diversity differed between LFF and HFF pigs (*p* = 0.001), and MFF and HFF pigs (*p* = 0.009). Beta diversity tended to differ between LFF and MFF pigs (*p* = 0.095) at this time. At dpi 42, no differences in alpha diversity were observed among treatments (*p* ≥ 0.199 for all contrasts). Beta diversity at dpi 42 differed between LFF and HFF pigs (*p* = 0.003), as well as MFF and HFF pigs (*p* = 0.036). Beta diversity at dpi 42 tended to differ between LFF and HFF pigs (*p* = 0.096).

**Figure 5:**
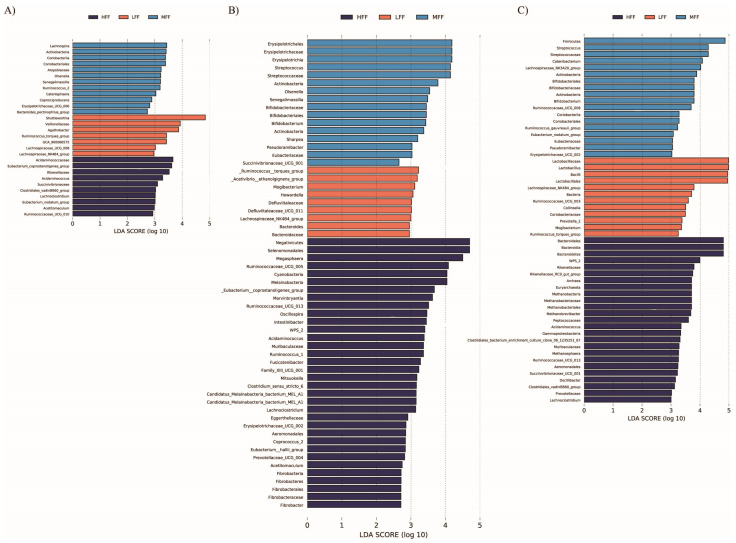
Annotated results from linear discriminant analysis in microbiota samples from pigs fed a lowly fermentable diet (LFF), an intermediate fermentability diet (MFF), and a highly fermentable diet (HFF) and inoculated with *Brachyspira hyodysenteriae*. All pigs were inoculated with *B. hyodysenteriae* at days post inoculation (dpi) 0 and fecal microbiota was assessed at (**A**) dpi 0, (**B**) dpi 12, and (**C**) dpi 42.

At the phylum level, abundance of Actinobacteria, Cyanobacteria, and Spirochaetes increased in more highly fermentable diets at dpi 0 (*p* = 0.029, *p* = 0.018, and *p* = 0.093, respectively; Table S2). At dpi 0, a number of bacterial genera differed significantly among dietary treatment groups, including *Olsenella*, *Acidaminococcus*, *Mitsuokella*, and a genus of *Succinovibrionaceae*, which were increased in HFF diets. Conversely, abundance of *Shuttleworthia* and a genus of *Veillonellaceae* were increased in LFF diets, both of which had high biological significance according to LEfSE analysis (Figure 5).

At dpi 12, Actinobacteria were reduced in LFF pigs (*p* = 0.024; Table S3), while Cyanobacteria, Fibrobacteres, and Eremiobacterota (WPS.2) were increased in HFF pigs (*p* = 0.021, *p* = 0.013, and *P* = 0.021, respectively). At the genus level, significantly differing genera included *Alloprevotella*, a genus of *Lachnospiraceae*, and *Shuttleworthia*, all of which were elevated in LFF pigs. Additionally, *Mogibacterium* was highly predictive for LFF pigs (linear discriminant analysis (LDA) score > 3) and *Megasphaera* was highly predictive for HFF pigs (LDA > 4). In MFF pigs, *Streptococcus* was greatly increased, with an LDA score over 4.0. At dpi 42, Actinobacteria, Bacteroidetes, and Firmicutes abundance were significantly different among treatments, while Cyanobacteria abundance tended to be greater in diets of higher fermentability (Table S4). Actinobacteria abundance was greater in higher fermentability diets, being the greatest in MFF pigs. Abundance of Bacteroidetes was the greatest in HFF pigs, while abundance of Firmicutes was lowest in HFF pigs. *Lactobacillus* abundance was highly associated with LFF pigs, having an LDA score approaching 5.0 (Figure 5). Additionally, *Mogibacterium* and *Prevotella_2* were also predictive for LFF pigs, while *Methanobrevibacter* and *Acidaminococcus* were highly predictive for HFF pigs.

**Figure 6:**
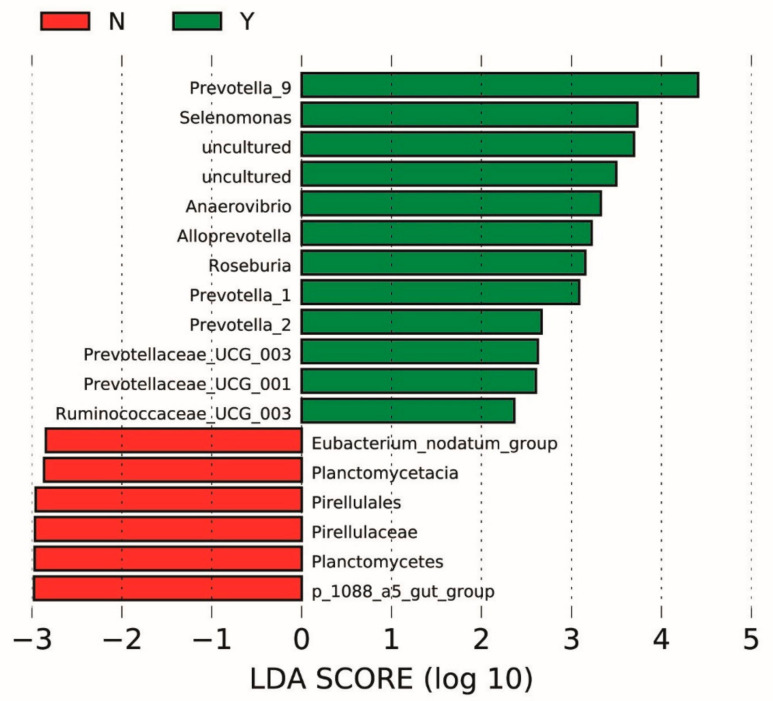
Predicted biological effect sizes of differential taxa using linear discriminant analysis (LDA) in the fecal microbiome of pigs at days post inoculation (dpi) 0 that either did (Y) or did not (N) develop clinical swine dysentery in the 42 d following inoculation with *Brachyspira hyodysenteriae*. The histogram of LDA scores calculated by LEfSe reveals differentially abundant taxa in the fecal microbiome of the two groups (red = N; green = Y) including overabundance of *Prevotella* spp. in pigs that developed clinical swine dysentery after inoculation.

Linear discriminant analysis also revealed differentially abundant taxa at dpi 0 that were associated with pigs that later developed clinical swine dysentery after experimental challenge, regardless of diet. (Figure 6) The fecal microbiota of pigs that eventually developed swine dysentery was enriched with members of the family Prevotellaceae, including *Alloprevotella*, *Prevotellaceae_UCG_003*, *Prevotellaceae_UCG001*, and three genera of *Prevotella*. Conversely, pigs that did not develop swine dysentery had microbiota containing members of the Planctomycetes phylum.

## 4. Discussion

Dietary manipulation to mitigate or prevent disease in pigs provides an attractive alternative to antibiotic usage. In particular, modifying diet fermentability changes the indigenous microbiota of the large intestine, which may alter susceptibility to bacterial diseases. *Brachyspira hyodysenteriae* is a large intestinal bacterial pathogen of which diet fermentability is known to influence colonization and disease susceptibility. However, inconsistent results have been reported due to differences in fiber type and composition of the basal diet [2,3,6,27]. Pigs fed highly digestible diets formulated with cooked white rice have increased large intestinal pH, reduced volatile fatty acid concentrations, and reduced clinical disease following *B. hyodysenteriae* challenge [5,27], suggesting that limiting large intestinal fermentation is preventative against disease. Additionally, cannulated pigs fed a nitrogen free diet based in cornstarch had reduced clinical disease and faster disease resolution following *B. hyodysenteriae* challenge, supporting the notion that highly digestible, lowly fermentable diets are beneficial to mitigate swine dysentery [28]. However, the protective effect of white rice has not always been observed, as parboiled rice did not mitigate disease [29]. These differences may be due to differences in rice processing, as cooked rice is readily digested in the small intestine while parboiled rice acts as a resistant starch, forgoing small intestinal digestion and undergoing large intestinal fermentation [30]. In pigs fed a corn-soybean meal-based diet, addition of 30% DDGS was found to exacerbate SD, suggesting that highly insoluble fibers in particular put pigs at greater risk to develop swine dysentery [4].

In addition to this work, several researchers have found that certain fibrous components provide protection from swine dysentery. Diets containing highly fermentable carbohydrates, specifically fructans provided as inulin, delayed onset and reduced the number of pigs that developed swine dysentery upon experimental challenge [3,6]. Although the differences between these previous studies make it difficult to draw universal conclusions, it seems that limiting insoluble, lowly fermentable fibers and increasing highly fermentable fibers could mitigate disease caused by *B. hyodysenteriae*. The fiber found in corn DDGS is mainly insoluble and poorly fermented, with the primary non-starch polysaccharides consisting of arabinoxylan and cellulose. Sugar beet pulp consists of more soluble and highly fermentable fibers, mainly from pectin [31]. Similarly, resistant starch is a type of carbohydrate similar to fiber and is defined by its resistance to degradation in the small intestine, but is highly fermented in the large intestine [32]. Thus, this study used highly fermentable sugar beet pulp and resistant potato starch in replacement of corn DDGS to evaluate whether a highly fermentable diet could mitigate swine dysentery.

Both longitudinal growth performance and clinical disease observations demonstrate a clear protection from disease in highly fermentable diets. Further, a graded response was observed, with the diet of intermediate fermentability providing some protection from disease, while pigs consuming the highest fermentability diet had near absence of clinical disease despite becoming colonized with the organism. The effect on growth performance was particularly striking in the first 21 dpi, when LFF pigs had clear growth curve deviations resulting from the challenge, MFF pigs had only a small growth curve deviation, and growth of HFF pigs remained linear throughout the 42 day challenge. This resulted in a 21.6 kg spread in dpi 42 body weights, demonstrating clear production relevance. We also observed reductions in digesta pH and an increase in core body temperatures as diet fermentability increased, both of which support increased large intestinal fermentation. As we were able to successfully demonstrate differences in diet fermentability and disease mitigation by the HFF diet, our next aim was to examine potential mechanisms by which this diet causes its protective effect.

One hypothesis for how diet may influence *B. hyodysenteriae* colonization and disease progression is via altered mucus production. The mucus layer is a critical component of the intestinal epithelium responsible for lubrication and protecting the epithelium from physical, chemical, enzymatic, and bacterial factors that could negatively impact animal health [7]. Dietary fiber is known to increase mucus secretion, with rats fed 5% insoluble citrus fiber having increased mucin production after 4 weeks [33]. In addition to modulating the quantity of mucus secreted, fibrous components can alter the composition of mucins produced. Secreted mucins are categorized as either neutral or acidic based on the composition of their oligosaccharide chains. Acidic mucins are further classified as sulfomucins or sialomucins, if the oligosaccharide contains either sulfate or sialic acid residues, respectively [34]. In a healthy pig, the colon is dominated by sulfomucins, but pigs clinically affected with swine dysentery have a marked increase in sialomucins [35]. Further, *B. hyodysenteriae* demonstrates chemotaxis toward sialated mucins [9], thus it is plausible differences in production of sialic acid alter susceptibility to *B. hyodysenteriae* challenge. Rats fed diets containing highly fermentable inulin had a predominance of sulfomucins in the colon, while those on a standard commercial diet had a greater proportion of sialomucins [36]. In the current study, no differences in sialomucins were observed. However, barring a few pigs that were still clinical for swine dysentery at the time of necropsy, sialomucins were absent or only present in very low abundance, consistent with what is observed in healthy pigs [35]. Interestingly, colonic sulfomucin-positive goblet cells, and thus total goblet cells, were lowest in pigs fed diets of high fermentability. Although this may suggest that the HFF diet reduces mucin secretion and that may reduce susceptibility to swine dysentery, these colon samples were collected at the end of the 42 day challenge, so the increased goblet cells in LFF pigs may have been a consequence of more of these pigs developing swine dysentery during the challenge period.

Diet-induced shifts in the indigenous microbiota have also been associated with altered *B. hyodysenteriae* susceptibility. However, a clear link between dietary mitigation of *B. hyodysenteriae* and microbial profiles has not been formed. Some interaction of *B. hyodysenteriae* with commensal bacteria is required, as gnotobiotic pigs fail to develop disease [37]. Specifically, other obligate anaerobes such as *Bacteroides vulgatus, Fusobacterium necrophorum*, and some *Clostridium* species have been shown to be fulfill this role and facilitate the development of swine dysentery [37,38]. Inoculated pigs that do or do not develop clinical swine dysentery also have distinct microbial patterns. Pigs that do not develop clinical disease have been associated with *Lactobacillus*, *Bifidobacterium*, and *Roseburia*, and those with clinical disease have been associated with *Desulfovibrio*, *Campylobacter*, *Mogibacterium*, and *Fusobacterium* [15]. Interestingly, the current experiment was unable to detect many OTUs consistent with *Fusobacterium* at any point in the study. Further, *Brachyspira* spp. were not identified at any point in the study, despite nearly 70% of pigs being positive for *Brachyspira hyodysenteriae* via culture of fecal swabs at the second collection (dpi 12). Other researchers with different experiment workflows have also had problems detecting *Brachyspira* spp. by 16S rRNA sequencing [39,40], so it seems 16S rRNA from this organism is poorly amplified by universal primers. To confirm this, we performed 16S rRNA sequencing directly on an aliquot of challenge inoculum and again there was no detection of *Brachyspira* spp. rRNA whereas next generation sequencing on this same aliquot of challenge inoculum contained abundant reads consistent with *B. hyodysenteriae*. This suggests that failure to detect *B. hyodysenteriae* in the 16 s analysis in the current study was due to a failure of amplification by the universal primers utilized. To investigate this further, we then made use of the Ribosomal Database Project’s Probe Match tool [41], and found the universal primers utilized herein do not align with *B. hyodysenteriae*, nor any other members of the *Brachyspiraceae* family. Although this retrospective finding is a limitation for the study herein, it demonstrates that bias can be introduced by the selection of 16S rRNA primers and that it may be necessary to pre-screen 16S rRNA universal primers when interested in specific bacterial taxa, such as during pathogen challenge studies or when attempting to enrich specific taxa with prebiotics or direct fed microbial probiotics.

The failure to detect *B. hyodysenteriae* also highlights the fact that 16S rRNA universal primers are not truly “universal” and fail to detect many bacterial communities depending on the sequence of the primer set used. In fact, it has been estimated that any single combination of PCR primers and DNA extraction techniques results in over half of bacterial taxa going undetected, regardless of sequencing depth [42]. This must be taken into account when making comparisons across studies, as differing primer sequences alone introduces large differences among datasets [43]. Further, assigning taxonomy based on different OTU databases, such as the Greengenes database used in Burrough et al. [15] compared with the SILVA database utilized herein, may also impact comparisons across studies [44]. Additional to these discrepancies, many other variables differ between studies, including inherent variation in fecal microbial populations, dietary adaptation time, subtle changes in specific fiber types, pig age, and pig genetics, to name a few. Although these limitations must be taken into consideration when making comparisons between different studies, this technology produces highly accurate semi-quantitative taxa counts to samples subjected to the same sequencing protocol [43]. Thus, despite the issues encountered, the current dataset allows for valid comparisons among dietary treatments within the current study, as all samples were subject to the same workflow.

Firmicutes and Bacteroidetes are consistently regarded as the primary phyla across microbiota studies. Previous work has shown increased Firmicutes:Bacteroidetes ratios in colonic contents of pigs with SD compared with inoculated pigs that did not develop SD [15]. In the current experiment, the lowly fermentable diet increased the ratio of Firmicutes:Bacteriodetes compared with pigs on more highly fermentable diets, a difference that was exacerbated as pigs developed clinical swine dysentery at dpi 12. In contrast to this work, pigs fed 30% lowly fermentable DDGS had lower ratios of Firmicutes:Bacteriodetes in the colon contents than those fed no DDGS [14]. However, Bacteriodetes were the dominant phyla in the DDGS study and colonic microbiota was evaluated, whereas Firmicutes were the primary phyla found in the fecal microbiome of the study herein, thus, a direct comparison is difficult to be made. Regardless, there does seem to be an association between greater relative Firmicutes abundance, lowly fermentable diets, and clinical swine dysentery that deserves further investigation.

Different types of dietary fiber have been shown to induce changes to microbial genera. However, results are highly variable. As there is still much we do not understand regarding specific microbial species and their preferred substrate, it is likely that specific fiber type and composition of base diet are important factors to enrich specific microbial genera and species. Weaned pigs fed 5% resistant potato starch had increases in fecal bacterial OTUs belonging to groups such as the genus *Megasphaera* and the family *Ruminococcaceae*, this corresponded with increased fecal concentrations of butyrate, caproate, and total volatile fatty acids [13]. Conversely, diets containing highly fermentable sugar beet pulp have been associated with reductions in *Ruminococcaeae* in the fecal microbiota of nursery pigs, highlighting the importance of differentiating between specific fiber types [45]. We did observe a genus of *Ruminococcaceae* to be associated with the HFF pigs at dpi 0, and *Megasphera* was highly predictive for HFF pigs at dpi 12, although this time point may have been confounded by differences in clinical disease presentation.

In the current study, *Shuttleworthia* was highly associated with the LFF diet at all time points, with an LDA score near 5.0 and relative abundance over 14% at dpi 0. *Shuttleworthia* are Firmicutes that belong to the family *Lachnospiraceae*, and are obligate anaerobes that mainly produce butyrate via fermentation of non-digestible carbohydrates [46]. Interestingly, *Shuttleworthia* prevalence is associated with male broilers of high body weight [47] and is reduced in calves fed a butyrate-fortified milk replacer [48]. Further, *Shuttleworthia* is reduced in the rumen of goats fed a high-grain diet compared with a high hay diet [49]. In ruminants, cereal grain diets provide rumen bacteria highly fermentable substrates, which are associated with a drop in pH and accumulation of volatile fatty acids, whereas a hay diet provides slow fermenting fibers primarily in the form of cellulose to rumen bacteria [49]. Similarly, the fiber provided by corn DDGS is primarily cellulose, thus the enrichment of *Shuttleworthia* in LFF pigs suggests that this bacterium may play a role in fermentation of insoluble fibers such as cellulose. *Shuttleworthia* has also been associated with both dental caries and periodontal disease in the human oral cavity, suggesting it has the potential to play a role in development of dysbiosis [50,51]. Additionally, it is an obligate anaerobe, as are many of the other microbial species implicated to have exacerbate SD [15,38]. The current study also observed other obligate anaerobes to be associated with the LFF diet, including *Agathobacter* (dpi 0), *Ruminococcus torques* (dpi 0) *Mogibacterium* (dpi 12), *Howardella* (dpi 12), and *Defluviitaleaceae* (dpi 12), furthering the hypothesis that certain anaerobic bacteria are associated with the development of SD [52,53,54]. These bacteria may act synergistically with *B. hyodysenteriae*, or perhaps a luminal environment that promotes the expansion of these anaerobes also promotes the colonization and replication of *B. hyodysenteriae.* It is also possible that some of the end-point metabolites of these bacterial populations could play a role in *B. hyodysenteriae*’s ability to associate with and replicate at the intestinal epithelium, relationships which warrant further investigation.

Regardless of treatment, *Prevotella* spp. were highly associated with pigs that developed clinical swine dysentery. *Prevotella* is widely regarded as a beneficial microbe. However, certain *Prevotella* spp. have been implicated as pathobionts and may promote inflammation, a classic feature of swine dysentery [55]. Interestingly, *Prevotella* spp. were found to be elevated in the colonic microbiota of pigs fed high levels of lowly fermentable DDGS, which has been shown to increase susceptibility to *B. hyodysenteriae* challenge. Association between *Prevotella* spp. and dietary fiber, particularly insoluble dietary fiber, has been observed previously in piglets supplemented with 1% lignocellulose [56]. This same study also observed that 1% inulin reduces *Prevotellaceae* abundance compared with piglets fed either 1% insoluble fiber or a standard corn soy diet [56]. Similarly, humans enrolled in a placebo-controlled study had reduced *Prevotella* abundance when given inulin compared with placebo [57], suggesting the type of dietary fiber may be important. In the current experiment, abundance of *Prevotella* spp. did not differ among dietary treatments at dpi 0, *Alloprevotella* was increased in LFF pigs at dpi 12, although this may be confounded by the fact that more of these pigs had clinical disease at the time of collection. Regardless, the association between *Prevotella* spp. and development of swine dysentery is striking, and there does appear to be an association with fiber level that could impact *B. hyodysenteriae* pathogenesis. As a great deal of genetic diversity exists among *Prevotella* species [58], it is possible only certain species and subtypes play a role in development of swine dysentery, relationships which should be investigated further. Interestingly, *Prevotella* species contain a glycosulphatase, which removes sulfate residues from mucins as the first step in mucus degradation, potentially allowing *B. hyodysenteriae* to associate with the epithelium easily [59]. In the current experiment, we observed another bacterium with mucin-degrading activity, *Ruminococcus torques* [60], to be highly associated with LFF pigs at dpi 0. This result, in combination with previous work, suggests that mucolytic bacteria such as *Prevotella, Ruminococcus torques*, and *Bacteroides vulgatus* are important for development of SD, and that perhaps high fiber, lowly fermentable ingredients such as DDGS promote expansion of these synergistic bacteria [15,38,60].

## 5. Conclusions

Taken together, feeding pigs more highly fermentable diets provided near complete protection from SD following experimental challenge with *B. hyodysenteriae*. Further, these diets were able to maintain growth rates and feed efficiency over *B. hyodysenteriae* challenged pigs fed diets with lower fermentability. This did not appear to be driven by differences in *B. hyodysenteriae* colonization, as fecal shedding of organism approached 90% across all treatments. To explain these findings, we performed fecal microbiota analysis at dpi 0, 12, and 42. The results of 16S rRNA sequencing highlighted the drawbacks of universal primers and the importance of pre-screening primers prior to sequencing, particularly when specific bacterial taxa are of interest. Regardless, the lowly fermentable diet, in which nearly all pigs developed swine dysentery, had several distinct microbial patterns including anaerobes such as *Shuttleworthia*, *Ruminococcus torques*, and *Mogibacterium*, which may potentially play a role in exacerbating disease. Further, enrichment of mucolytic bacteria in pigs fed the lowly fermentable diet suggests that these bacteria may also aid in colonization and development of disease. It is likely that either these bacteria directly or their end-point metabolites provide a niche favorable for *B. hyodysenteriae* growth, leading to a greater incidence of disease.

## Figures and Tables

**Table 1:** Diet composition, as fed.

Ingredient, %	LFF	MFF	HFF
Corn	57.68	55.20	51.00
Soybean meal	18.40	20.60	23.70
Soybean oil	1.00	1.27	2.37
Salt	0.35	0.35	0.35
Monocalcium phosphate, 21%	0.63	0.78	0.94
Limestone	1.24	1.03	0.85
L-lysine HCl	0.36	0.34	0.29
L-threonine	0.04	0.07	0.09
DL-methionine	-	0.06	0.11
Vitamin premix ^1^	0.15	0.15	0.15
Trace mineral premix ^2^	0.15	0.15	0.15
Corn DDGS ^3^	20.00	10.00	-
Resistant potato starch	-	5.00	10.00
Sugar beet pulp	-	5.00	10.00
*Calculated composition*			
Metabolizable energy, kcal/kg	3341	3300	3301
Crude protein, %	19.36	17.97	16.82
SID lysine ^4^, %	0.98	0.98	0.98
SID methionine + cysteine, %	0.55	0.55	0.56
SID threonine, %	0.60	0.60	0.58
SID tryptophan, %	0.16	0.16	0.16
*Analyzed composition*			
DM, %	88.5	88.6	88.8
Crude protein, %	17.19	16.72	14.72
Gross energy, kcal/kg	3957	3884	3875
Acid detergent fiber, %	4.47	4.31	4.24
Neutral detergent fiber, %	11.93	9.52	8.30
Insoluble dietary fiber, %	13.56	10.99	8.94
Total dietary fiber, %	13.95	11.33	9.20
Resistant starch	3.45	6.83	10.02

^1^ Provided per kilogram of diet: 6125 IU vitamin A, 700 IU vitamin D_3_, 50 IU vitamin E, 30 mg vitamin K, 0.05 mg vitamin B_12_, 11 mg riboflavin, 56 mg niacin, and 27 mg pantothenic acid. ^2^ Provided per kilogram of diet: 22 mg Cu (as CuSO_4_), 220 mg Fe (as FeSO_4_), 0.4 mg I (as Ca(IO_3_)_2_), 52 mg Mn (as MnSO_4_), 220 mg Zn (as ZnSO_4_), and 0.4 mg Se (as Na_2_SeO_3_). ^3^ DDGS = distiller’s dried grains with solubles. ^4^ SID = standardized ileal digestibility.

**Table 2:** Incidence of clinical disease of pigs inoculated with *Brachyspira hyodysenteriae* and fed a lowly fermentable diet (LFF), an intermediate fermentability diet (MFF), and a highly fermentable diet (HFF).

	Treatment ^1^			*p*-Value
	LFF	MFF	HFF	
Number of pigs with clinical disease ^2^	11/13	6/13	2/13	0.002
Median days to clinical disease ^2^	9	20	-	<0.001
Median days with clinical disease ^2^	6	2	0	0.001
Number of pigs positive ^3^	12/13	12/13	11/13	0.588

^1^ n = 13 pigs/treatment. ^2^ Clinical SD = diarrhea containing both blood and mucus. ^3^ Fecal positive via selective anaerobic culture at any point during the 42 day study. Fecal swabs were collected and cultured for B. hyodysenteriae at days post inoculation 4, 8, 12, 14, 15, 20, 28, 35, and 42.

**Table 3:** Weekly growth performance of pigs inoculated with *Brachyspira hyodysenteriae* and fed a lowly fermentable diet (LFF), an intermediate fermentability diet (MFF), and a highly fermentable diet (HFF).

Item	Treatment ^1^			SEM	*p*-Value
	LFF	MFF	HFF		
Pre-Challenge (dpi −14 to 0)					
ADG, kg/d	0.79	0.84	0.89	0.039	0.197
ADFI, kg/d	1.83	1.84	1.74	0.068	0.506
G:F	0.43 ^b^	0.46 ^b^	0.50 ^a^	0.012	0.001
dpi 0 to 7					
ADG, kg/d	1.12 ^b^	1.37 ^a^	1.33 ^a^	0.056	0.007
ADFI, kg/d	2.33	2.43	2.24	0.075	0.221
G:F	0.50 ^b^	0.57 ^a^	0.59 ^a^	0.018	0.002
dpi 8 to 14					
ADG, kg/d	−0.48 ^b^	0.38 ^a^	1.10 ^a^	0.231	<0.001
ADFI, kg/d	1.00 ^b^	2.06 ^a^	2.30 ^a^	0.212	<0.001
G:F	−1.97 ^b^	0.02 ^a^	0.44 ^a^	0.422	0.001
dpi 15 to 21					
ADG, kg/d	0.63	0.61	1.14	0.249	0.232
ADFI, kg/d	1.39 ^b^	1.96 ^ab^	2.49 ^a^	0.261	0.014
G:F	0.32	0.33	0.40	0.149	0.703
dpi 22 to 28					
ADG, kg/d	1.25	1.04	1.13	0.158	0.625
ADFI, kg/d	2.41	2.24	2.79	0.250	0.277
G:F	0.53	0.43	0.40	0.062	0.233
dpi 29 to 35					
ADG, kg/d	1.34	1.36	1.48	0.106	0.562
ADFI, kg/d	2.97	3.00	3.12	0.167	0.766
G:F	0.42	0.48	0.49	0.053	0.558
dpi 36 to 42					
ADG, kg/d	0.97 ^b^	1.41 ^a^	1.33 ^a^	0.129	0.033
ADFI, kg/d	2.88 ^b^	3.57 ^a^	3.48 ^a^	0.172	0.009
G:F	0.33	0.40	0.38	0.043	0.458
dpi 0 BW, kg	40.0	40.6	41.9	1.410	0.623
dpi 42 BW, kg	73.0 ^b^	83.3 ^b^	94.6 ^a^	3.150	<0.001

^1^ n = 13 pigs/treatment. ^a–c^ Means with differing superscripts differ significantly at *p* < 0.05. dpi = day post inoculation.

**Table 4:** Digesta pH at days post inoculation 42 of pigs inoculated with *Brachyspira hyodysenteriae* and fed a lowly fermentable diet (LFF), an intermediate fermentability diet (MFF), and a highly fermentable diet (HFF).

Item	Treatment ^1^			SEM	*p*-Value
	LFF	MFF	HFF		
Cecum pH	5.92 ^a^	5.71 ^b^	5.68 ^b^	0.058	0.013
Colon pH	6.71	6.59	6.56	0.056	0.139

^1^ n = 13 pigs/treatment. ^a–c^ Means with differing superscripts differ significantly at *p* < 0.05.

**Table 5:** Sulfomucin- and sialomucin-positive goblet cells in the colon at 42 days post inoculation of pigs inoculated with *Brachyspira hyodysenteriae* and fed a lowly fermentable diet (LFF), an intermediate fermentability diet (MFF), and a highly fermentable diet (HFF).

Item	Treatment ^1^			SEM	*p*-Value
	LFF	MFF	HFF		
Sulfomucin-positive goblet cells/10,000 μm^2^	12.5 ^a^	10.0 ^a,b^	9.0 ^b^	0.979	0.034
Sialomucin-positive goblet cells/10,000 μm^2^	0.04	0.05	0.05	0.066	0.990

^1^ n = 13 pigs/treatment. ^a–c^ Means with differing superscripts differ significantly at *p* < 0.05.

## Data Availability

Raw sequence data are available in the Sequence Read Archive (accession #PRJNA678760). All other data presented in this study are available within the article and its Supplementary Materials.

## Supplementary Materials

The following are available online at https://www.mdpi.com/2076-2615/11/2/396/s1, Table S1: Summary of fecal selective anaerobic culture results of pigs inoculated with *Brachyspira hyodysenteriae* and fed a lowly fermentable diet (LFF), an intermediate fermentability diet (MFF), and a highly fermentable diet (HFF) wherein “0” denotes a negative culture outcome and “+” denotes a positive culture outcome, Table S2: Relative abundance of bacterial phyla between pigs inoculated with *Brachyspira hyodysenteriae* and fed a lowly fermentable diet (LFF), an intermediate fermentability diet (MFF), and a highly fermentable diet (HFF) at days post inoculation 0, Table S3: Relative abundance of bacterial phyla between pigs inoculated with *Brachyspira hyodysenteriae* and fed a lowly fermentable diet (LFF), an intermediate fermentability diet (MFF), and a highly fermentable diet (HFF) at days post inoculation 12, Table S4: Relative abundance of bacterial phyla between pigs inoculated with *Brachyspira hyodysenteriae* and fed a lowly fermentable diet (LFF), an intermediate fermentability diet (MFF), and a highly fermentable diet (HFF) at days post inoculation 42, Table S5: Relative abundance of bacterial genera that differ significantly between pigs prior to inoculation with *Brachyspira hyodysenteriae* and fed a lowly fermentable diet (LFF), an intermediate fermentability diet (MFF), and a highly fermentable diet (HFF) at days post inoculation 0, Table S6: Relative abundance of bacterial genera that differ significantly between pigs inoculated with *Brachyspira hyodysenteriae* and fed a lowly fermentable diet (LFF), an intermediate fermentability diet (MFF), and a highly fermentable diet (HFF) at days post inoculation 12. Table S7: Relative abundance of bacterial genera that differ significantly between pigs inoculated with *Brachyspira hyodysenteriae* and fed a lowly fermentable diet (LFF), an intermediate fermentability diet (MFF), and a highly fermentable diet (HFF) at days post inoculation 42, Figure S1: Daily core body temperatures of pigs inoculated with *Brachyspira hyodysenteriae* and fed a lowly fermentable diet (LFF), an intermediate fermentable diet (MFF), and a highly fermentable diet (HFF). Pigs were inoculated at days post inoculation (dpi) 0 and assessed each morning for 42 dpi. Data represents 13 pigs per treatment.
