# Supplementary material for: Highly Fermentable Fiber Alters Fecal Microbiota and Mitigates Swine Dysentery Induced by Brachyspira hyodysenteriae
Source: Animals (Basel). 2021 Feb 4;11(2):396. doi: 10.3390/ani11020396 (PMC7915590; doi:10.3390/ani11020396)
Supplement: Supplementary file 1 [file animals-11-00396-s001.pdf]

animals

Article

Highly Fermentable Fiber Alters Fecal Microbiota and Mitigates Swine Dysentery Induced by *Brachyspira hyodysenteriae*

Emma T. Helm [1], Nicholas K. Gabler [1] and Eric. R. Burrough [2,*]

[1] Department of Animal Science, Iowa State University, Ames 50011, IA, USA; ethelm@iastate.edu (E.T.H.); ngabler@iastate.edu (N.K.G.)
[2] Department of Veterinary Diagnostic and Production Animal Medicine, Iowa State University, Ames 50011, IA, USA
* Correspondence: burrough@iastate.edu; Tel.: +1515-294-1950

Citation: Helm, E.T.; Gabler, N.K.; Burrough, E.R. Highly Fermentable Fiber Alters Fecal Microbiota and Mitigates Swine Dysentery Induced by *Brachyspira hyodysenteriae*. *Animals* **2021**, *10*, x. https://doi.org/10.3390/ani11020396

Academic Editor: M. Ángeles Latorre
Received: 14 January 2021
Accepted: 2 February 2021
Published: 4 February 2021

Publisher's Note: MDPI stays neutral with regard to jurisdictional claims in published maps and institutional affiliations.

Copyright: © 2021 by the authors. Licensee MDPI, Basel, Switzerland. This article is an open access article distributed under the terms and conditions of the Creative Commons Attribution (CC BY) license (http://creativecommons.org/licenses/by/4.0/).

Table S1. Summary of fecal selective anaerobic culture results of pigs inoculated with *Brachyspira hyodysenteriae* and fed a lowly fermentable diet (LFF), an intermediate fermentability diet (MFF), and a highly fermentable diet (HFF) wherein "0" denotes a negative culture outcome and "+" denotes a positive culture outcome.

Pig	TRT [1]	dpi –9	dpi 4	dpi 8	dpi 12	dpi 16	dpi 20	dpi 28	dpi 35	dpi 42
69	LFF	0	0	0	0	0	+	+	+	0
66	LFF	0	0	+	+	+	+	0	0	+
43	LFF	0	0	+	+	0	+	+	+	+
25	LFF	0	0	+	+	+	0	0	0	0
47	LFF	0	0	0	0	0	0	0	0	0
15	LFF	0	0	+	+	+	+	+	0	0
34	LFF	0	0	0	0	+	+	0	0	+
41	LFF	0	0	+	+	+	+	+	0	0
49	LFF	0	0	+	0	+	+	+	0	+
13	LFF	0	0	+	+	0	0	0	0	0
35	LFF	0	0	+	+	+	0	0	+	+
46	LFF	0	0	0	+	+	+	+	+	0
45	LFF	0	0	+	+	0	0	0	+	0
23	MFF	0	0	0	0	0	+	+	+	0
17	MFF	0	0	0	0	0	+	0	+	0
48	MFF	*	0	+	+	+	+	+	+	+
28	MFF	0	0	0	+	+	+	0	0	0
50	MFF	0	0	0	+	+	0	0	0	0
11	MFF	0	0	0	0	0	0	0	0	0
19	MFF	0	0	0	+	0	+	+	0	0
53	MFF	*	0	0	+	+	+	+	+	0
59	MFF	0	0	+	+	+	0	0	0	0
26	MFF	0	0	0	+	+	+	+	+	0
22	MFF	0	0	+	0	0	0	0	0	+
65	MFF	0	0	0	0	0	+	+	0	0
20	MFF	0	0	+						
29	HFF	0	0	+	+	0	+	0	0	+
62	HFF	0	0	0	0	0	0	0	0	0
57	HFF	0	0	0	+	0	0	0	+	0
51	HFF	0	0	0	0	0	0	0	0	0
63	HFF	0	0	0	0	0	0	0	0	0
58	HFF	0	0	0	0	0	+	0	0	+
60	HFF	*	0	0	+	+	+	+	+	0
55	HFF	0	0	+	0	0	0	+	0	+
21	HFF	0	0	0	0	0	+	+	+	0
39	HFF	0	0	+	+	0	0	0	+	+
24	HFF	0	0	0	0	+	+	+	0	0
33	HFF	0	0	0	+	0	+	+	0	0
44	HFF	0	0	0	+	0	0	0	+	0

[1] TRT = treatment. * positive for *B. murdochii.*

Table S2. Relative abundance of bacterial phyla between pigs inoculated with *Brachyspira hyodysenteriae* and fed a lowly fermentable diet (LFF), an intermediate fermentability diet (MFF), and a highly fermentable diet (HFF) at days post inoculation 0**.**

	Treatment [1]				
**Item**	**LFF**	**MFF**	**HFF**	**SEM**	***p*-Value [2]**
Actinobacteria	0.33	0.95	0.89	0.224	0.029
Bacteroidetes	10.1	13.3	10.2	4.018	0.803
Chlamydiae	0.003	0.007	0.007	0.006	0.803
Cyanobacteria	0.067	0.264	1.450	0.774	0.018
Fibrobacteres	0.000	0.003	0.044	0.047	0.655
Firmicutes	86.0	82.0	81.1	3.677	0.803
Planctomycetes	0.015	0.047	0.132	0.110	0.615
Proteobacteria	2.65	2.38	4.73	1.620	0.661
Spirochaetes	0.012	0.042	0.129	0.074	0.093
Tenericutes	0.825	0.914	1.170	0.447	0.803
WPS.2	0.003	0.000	0.010	0.023	0.803

[1] n = 10 pigs per treatment. [2] *p*-values were adjusted with a false discovery rate controlled at 5%.

Table S3. Relative abundance of bacterial phyla between pigs inoculated with *Brachyspira hyodysenteriae* and fed a lowly fermentable diet (LFF), an intermediate fermentability diet (MFF), and a highly fermentable diet (HFF) at days post inoculation 12**.**

	Treatment [1]				
**Item**	**LFF**	**MFF**	**HFF**	**SEM**	***p*-Value [2]**
Actinobacteria	0.373	1.534	1.042	0.005	0.024
Bacteroidetes	9.31	6.44	12.15	3.686	0.593
Chlamydiae	0.003	0.008	0.123	0.008	0.593
Cyanobacteria	0.324	0.276	2.247	1.000	0.021
Fibrobacteres	0.000	0.013	0.075	0.060	0.013
Firmicutes	86.4	87.6	79.0	3.238	0.424
Planctomycetes	0.261	0.208	0.363	0.209	0.903
Proteobacteria	1.181	1.406	1.777	0.559	0.878
Spirochaetes	0.357	0.329	0.248	0.222	0.909
Tenericutes	1.572	1.905	2.341	0.622	0.866
WPS.2	0.001	0.012	0.470	0.544	0.021
Firmicutes:Bacteroidetes	59.09	29.16	12.32	18.347	0.006

[1] n = 10 pigs per treatment. [2] *p*-values were adjusted with a false discovery rate controlled at 5%.

Table S4. Relative abundance of bacterial phyla between pigs inoculated with *Brachyspira hyodysenteriae* and fed a lowly fermentable diet (LFF), an intermediate fermentability diet (MFF), and a highly fermentable diet (HFF) at days post inoculation 42**.**

	Treatment [1]				
**Item**	**LFF**	**MFF**	**HFF**	**SEM**	***p*-Value [2]**
Actinobacteria	0.283	1.647	0.562	0.004	0.001
Bacteroidetes	10.56	6.88	20.12	4.389	0.019
Chlamydiae	0.003	0.002	0.006	0.003	0.405
Cyanobacteria	0.091	0.240	0.390	0.145	0.077
Fibrobacteres	0.000	0.000	0.001	0.022	0.138
Firmicutes	84.7	85.4	70.2	3.161	0.004
Planctomycetes	0.003	0.006	0.006	0.307	0.524
Proteobacteria	1.57	1.30	2.07	0.605	0.538
Spirochaetes	0.534	1.213	0.364	0.652	0.383
Tenericutes	1.42	2.00	2.58	0.607	0.322
WPS.2	0.201	0.067	1.774	1.814	0.149

Firmicutes:Bacteroidetes	18.60	29.18	5.36	8.358	0.001

[1] n = 10 pigs per treatment. [2] *p*-values were adjusted with a false discovery rate controlled at 5%.

Table S5. Relative abundance of bacterial genera that differ significantly between pigs prior to inoculation with *Brachyspira hyodysenteriae* and fed a lowly fermentable diet (LFF), an intermediate fermentability diet (MFF), and a highly fermentable diet (HFF) at days post inoculation 0**.**

**Item**		**Treatment** [1]			**SEM**	***p*-Value** [2]
		**LFF**	**MFF**	**HFF**		
Actinobacteria						
Atopobiaceae	*Olsenella*	0.034	0.488	0.436	0.276	0.038
Bacteroidetes						
Muribaculaceae	uncultured	0.095	0.116	0.614	0.206	0.013
Cyanobacteria						
Uncultured	*Candidatus.Melainabacteria.bacterium.MEL.A1*	0.000	0.083	0.055	0.075	0.012
Uncultured	uncultured	0.063	0.157	1.247	0.653	0.017
Firmicutes						
Clostridiales Family XIII	*Eubacterium nodatum group*	0.022	0.071	0.187	0.072	0.028
Lachnospiraceae	*Ruminococcus torques group*	0.536	0.150	0.004	0.310	0.001
Lachnospiraceae	*Acetitomaculum*	0.002	0.054	0.128	0.076	0.004
Lachnospiraceae	*Lachnospira*	0.000	0.008	0.003	0.275	0.004
Lachnospiraceae	*Lachnospiraceae NK3A20 group*	0.410	5.114	0.920	1.816	0.004
Lachnospiraceae	*Shuttleworthia*	14.52	6.15	1.11	5.348	0.004
Ruminococcaceae	*Eubacterium coprostanoligenes group*	0.217	0.746	0.990	0.246	0.008
Erysipelotrichaceae	*Catenisphaera*	0.035	0.209	0.027	0.053	0.001
Erysipelotrichaceae	*Erysipelotrichaceae UCG 002*	0.004	0.179	0.024	0.128	0.040
Erysipelotrichaceae	*Erysipelotrichaceae UCG 006*	0.000	0.002	0.000	0.062	0.014
Acidaminococcaceae	*Acidaminococcus*	0.021	0.222	0.358	0.156	0.004
Veillonellaceae	uncultured	1.846	0.622	0.129	0.698	0.004
Veillonellaceae	*Mitsuokella*	0.237	0.949	1.175	0.346	0.017
Proteobacteria						
Succinovibrionaceae	*Succinivibrionaceae*	0.01	0.009	0.162	0.107	0.046
Tenericutes						
Metagenome	*metagenome*	0.050	0.000	0.000	0.047	0.042
Uncultured	uncultured	0.013	0.034	0.000	0.011	0.038

[1]n = 10 pigs per treatment. [2] *p*-values were adjusted with a false discovery rate controlled at 5%.

Table S6. Relative abundance of bacterial genera that differ significantly between pigs inoculated with *Brachyspira hyodysenteriae* and fed a lowly fermentable diet (LFF), an intermediate fermentability diet (MFF), and a highly fermentable diet (HFF) at days post inoculation 12**.**

Item		Treatment [1]			SEM	*p*-Value [2]
		LFF	MFF	HFF		
	Actinobacteria					
Bifidobacteriaceae	*Bifidobacterium*	0.081	0.602	0.363	0.244	0.031
Atopobiaceae	*Olsenella*	0.014	0.644	0.41	0.377	0.006
	Bacteroidetes					
Muribaculaceae	uncultured	0.000	0.017	0.413	0.416	0.009
Muribaculaceae	*metagenome*	0.001	0.008	0.037	0.028	0.092
Muribaculaceae	uncultured	0.24	0.238	1.928	0.77	0.016
Muribaculaceae	*uncultured rumen bacterium*	0.046	0.245	0.572	0.349	0.099
Prevotellaceae	*Alloprevotella*	2.909	0.26	0.277	1.558	0.031
Prevotellaceae	*Prevotellaceae NK3B31 group*	0.046	0.266	0.74	0.388	0.025
Prevotellaceae	*Prevotellaceae UCG 004*	0.002	0.01	0.072	0.050	0.047
Rikenellaceae	*Rikenellaceae RC9 gut group*	3.641	0.581	1.209	1.494	0.073
Uncultured	uncultured	0.004	0.072	0.126	0.068	0.009
	Cyanobacteria					
Uncultured	uncultured	0.225	0.187	1.507	0.097	0.025
Uncultured	*uncultured rumen bacterium*	0.004	0.000	0.055	0.059	0.079
	Fibrobacteres					
Fibrobacteraceae	*Fibrobacter*	0.000	0.013	0.075	0.060	0.016
	Firmicutes					
Streptococcaceae	*Streptococcus*	0.449	3.168	0.937	1.382	0.073
Christensenellaceae	*Christensenellaceae R.7 group*	0.644	1.669	1.888	0.511	0.088
Clostridiaceae	*Clostridium sensu stricto 6*	1.322	1.120	2.843	1.005	0.018
Clostridiales vadinBB60 group	*uncultured rumen bacterium*	0.000	0.026	0.047	0.030	0.007
Clostridiales family XIII	*Eubacterium nodatum group*	0.017	0.053	0.358	0.175	0.014
Clostridiales family XIII	*Family XIII UCG 001*	0.057	0.221	0.347	0.117	0.022
Clostridiales family XIII	*Mogibacterium*	0.261	0.090	0.047	0.075	0.016
Lachnospiraceae	*Lachnospiraceae*	21.970	4.070	1.830	7.575	0.005
Lachnospiraceae	*Eubacterium ruminantium group*	0.124	0.375	4.128	1.958	0.005
Lachnospiraceae	*Acetitomaculum*	0.002	0.022	0.092	0.058	0.021
Lachnospiraceae	*Marvinbryantia*	0.159	0.156	0.927	0.340	0.021
Lachnospiraceae	*Shuttleworthia*	11.25	11.00	1.18	5.024	0.018
Peptostreptococcaceae	*Intestinibacter*	0.148	0.148	0.698	0.230	0.025
Ruminococcaceae	*Oscillibacter*	0.025	0.050	0.345	0.188	0.038
Ruminococcaceae	*Oscillospira*	0.073	0.133	0.569	0.228	0.027
Ruminococacceae	*Ruminococcaceae UCG 005*	1.337	1.657	3.548	0.858	0.096
Ruminococacceae	*Ruminococcaceae UCG 013*	3.313	3.916	2.663	0.886	0.018
Ruminococacceae	uncultured	0.142	0.415	0.554	0.148	0.025
Erysipelotrichaceae	*Holdemanella*	1.834	1.300	0.493	0.555	0.073
Erysipelotrichaceae	uncultured	2.369	0.117	0.135	1.076	0.005
	Proteobacteria					
Succinivibrionaceae	uncultured	0.000	0.031	0.004	0.029	0.031
Succinivibrionaceae	*Succinivibrio*	0.010	0.027	0.072	0.033	0.094
Succinivibrionaceae	*Succinivibrionaceae UCG 001*	0.005	0.077	0.054	0.036	0.016
Enterobacteriaceae	*Escherichia Shigella*	0.444	0.003	0.009	0.417	0.021
	WPS.2					
Uncultured	*uncultured rumen bacterium*	0.001	0.012	0.470	0.544	0.033

[1] n = 10 pigs per treatment. [2] *p*-values were adjusted with a false discovery rate controlled at 5%.

Table S7. Relative abundance of bacterial genera that differ significantly between pigs inoculated with *Brachyspira hyodysenteriae* and fed a lowly fermentable diet (LFF), an intermediate fermentability diet (MFF), and a highly fermentable diet (HFF) at days post inoculation 42.

Item		Treatment [1]			SEM	*p*-Value [2]
		LFF	MFF	HFF		
Archaea						
Methanobacteriaceae	*Methanobrevibacter*	0.158	0.633	1.156	0.39	0.015
Methanobacteriaceae	*Methanosphaera*	0.002	0.006	0.053	0.023	0.002
Actinobacteria						
Bifidobacteriaceae	*Bifidobacterium*	0.093	1.118	0.445	0.426	0.010
Atopobiaceae	*Olsenella*	0.009	0.32	0.019	0.248	0.056
Eggerthellaceae	uncultured	0.007	0.016	0.005	0.004	0.075
Eggerthellaceae	uncultured	0.05	0.041	0.017	0.013	0.097
Uncultured	uncultured	0.025	0.028	0.007	0.008	0.036
Bacteroidetes						
Muribaculaceae	uncultured	0.324	0.297	1.775	0.585	0.015
Muribaculaceae	*Uncultured Porphyromonadaceae bacterium*	4.278	2.707	10.13	3.046	0.083
Muribaculaceae	*uncultured rumen bacterium*	0.067	0.323	0.223	0.115	0.074
Prevotellaceae	uncultured	0.013	0.033	0.145	0.017	0.074
Rikenellaceae	*Rikenellaceae RC9 gut group*	0.591	0.617	1.727	0.467	0.081
Firmicutes						
Lactobacillaceae	*Lactobacillus*	23.61	9.904	3.731	5.716	0.003
Streptococcaceae	*Streptococcus*	0.508	3.673	2.081	1.563	0.064
Christensenellaceae	uncultured	0.000	0.007	0.012	0.007	0.036
Clostridiales vadin BB60 group	*D. Clostridiales bacterium enrichment culture clone 06.1235251.67*	0.000	0.000	0.003	0.026	0.074
Clostridiales vadin BB60 group	*uncultured rumen bacterium*	0.000	0.003	0.035	0.014	0.001
Eubacteriaceae	*Pseudoramibacter*	0.003	0.186	0.020	0.115	0.010
Clostridiales family XIII	*Eubacterium nodatum group*	0.006	0.084	0.019	0.039	0.023
Clostridiales family XIII	*Mogibacterium*	0.106	0.088	0.019	0.035	0.023
Lachnospiraceae	*Eubacterium hallii group*	0.079	0.286	0.106	0.084	0.074
Lachnospiraceae	*Ruminococcus gauvreauii group*	0.104	0.423	0.108	0.136	0.055
Lachnospiraceae	*Lachnospiraceae NK3A20 group*	0.575	2.415	0.205	0.828	0.005
Lachnospiraceae	*Lachnospiraceae NK4A136 group*	0.901	0.150	0.351	0.287	0.023
Lachnospiraceae	*Lachnospiraceae NK4B4 group*	0.054	0.014	0.002	0.031	0.015
Ruminococcaceae	*Ruminococcaceae UCG 008*	0.716	1.195	0.171	0.427	0.023
Ruminococcaceae	uncultured	0.391	0.542	1.213	0.119	0.036
Erysipelotrichaceae	*Catenibacterium*	0.181	2.638	0.206	1.309	0.015
Erysipelotrichaceae	*Catenisphaera*	0.060	0.194	0.044	0.015	0.064
Erysipelotrichaceae	*Erysipelotrichaceae UCG 002*	0.000	0.163	0.069	0.150	0.010
Acidaminococcaceae	*Acidaminococcus*	0.051	0.079	0.470	0.209	0.036
Proteobacteria						
Succinovibrionaceae	*Succinivibrionaceae UCG 001*	0.019	0.116	0.289	0.153	0.037

[1] n = 10 pigs per treatment. [2] *p*-values were adjusted with a false discovery rate controlled at 5%.

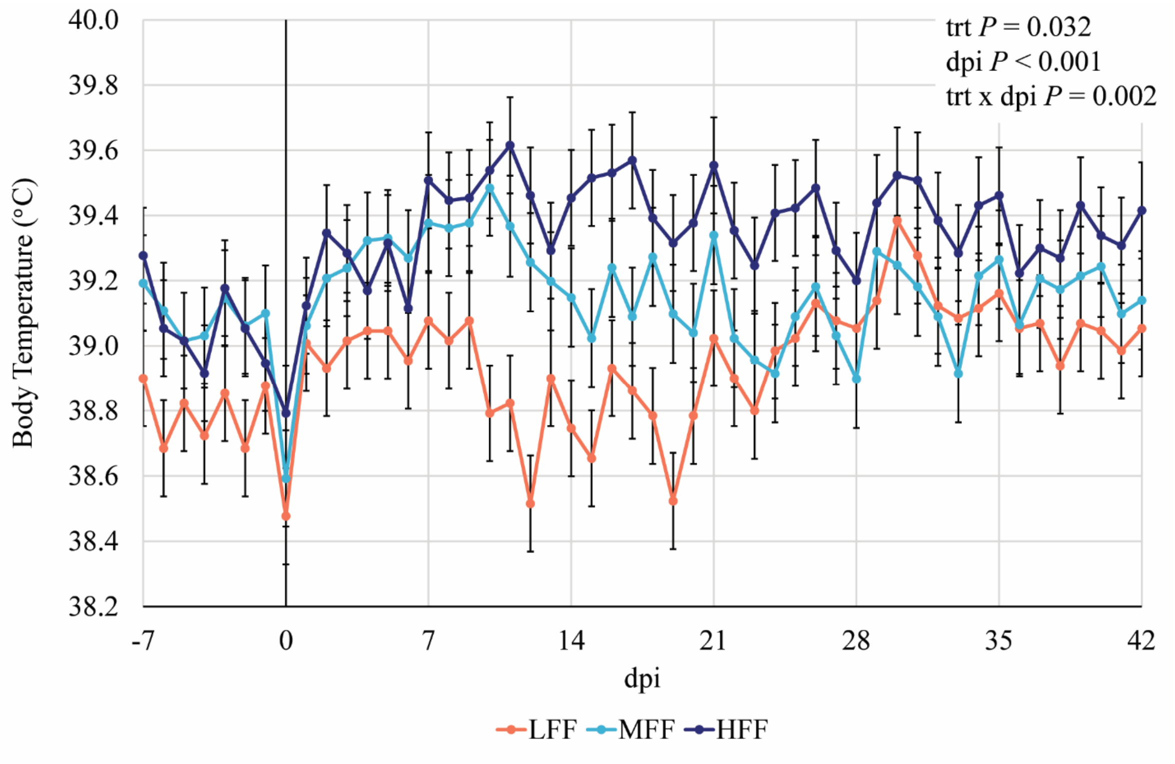

Figure S1. Daily core body temperatures of pigs inoculated with *Brachyspira hyodysenteriae* and fed a lowly fermentable diet (LFF), an intermediate fermentable diet (MFF), and a highly fermentable diet (HFF). Pigs were inoculated at days post inoculation (dpi) 0 and assessed each morning for 42 dpi. Data represents 13 pigs per treatment.
